# Supplementary material for: Mapping the evolution of fertility support policies in China: A content and instrumental analysis
Source: PLoS One. 2025 Oct 9;20(10):e0332137. doi: 10.1371/journal.pone.0332137 (PMC12510515; doi:10.1371/journal.pone.0332137)
Supplement: S1 Appendix — (ZIP) [file pone.0332137.s001.zip › S1 Appendix. 226 original policy documents/95-流动人口计划生育工作条例(FBM-CLI-2-117174).doc]

流动人口计划生育工作条例

**发布部门：** [国务院](https://www.pkulaw.com/law/chl?Aggs.IssueDepartment=5)

**发文字号：**中华人民共和国国务院令第555号

**发布日期：**2009.05.11

**实施日期：**2009.10.01

**时效性：** [失效](https://www.pkulaw.com/law/chl?Aggs.TimelinessDic=02)

**效力级别：** [行政法规](https://www.pkulaw.com/law/chl?Aggs.EffectivenessDic=XC0201)

**法规类别：** [计划生育管理](https://www.pkulaw.com/law/chl?Aggs.Category=09705)

**失效依据：**[国务院关于废止部分行政法规的决定(2021)](javascript:TMP(5077189,0,'chl'))

中华人民共和国国务院令
（第555号）

　　《流动人口计划生育工作条例》已经2009年4月29日国务院第60次常务会议通过，现予公布，自2009年10月1日起施行。

总　理　　温家宝
二○○九年五月十一日

流动人口计划生育工作条例

　　**第一条**　为了加强流动人口计划生育工作，寓管理于服务之中，维护流动人口的合法权益，稳定低生育水平，根据《[中华人民共和国人口与计划生育法](https://www.pkulaw.com/chl/252f7ce51132230bbdfb.html)》，制定本条例。

　　**第二条**　本条例所称流动人口，是指离开户籍所在地的县、市或者市辖区，以工作、生活为目的异地居住的成年育龄人员。但是，下列人员除外：

　　（一）因出差、就医、上学、旅游、探亲、访友等事由异地居住、预期将返回户籍所在地居住的人员；

　　（二）在直辖市、设区的市行政区域内区与区之间异地居住的人员。

　　**第三条**　县级以上地方人民政府领导本行政区域内流动人口计划生育工作，将流动人口计划生育工作纳入本地经济社会发展规划，并提供必要的保障；建立健全流动人口计划生育工作协调机制，组织协调有关部门对流动人口计划生育工作实行综合管理；实行目标管理责任制，对有关部门承担的流动人口计划生育工作进行考核、监督。

　　**第四条**　流动人口计划生育工作由流动人口户籍所在地和现居住地的人民政府共同负责，以现居住地人民政府为主，户籍所在地人民政府予以配合。

　　**第五条**　国务院人口和计划生育部门主管全国流动人口计划生育工作，制定流动人口计划生育工作规划并组织实施；建立流动人口计划生育信息管理系统，实现流动人口户籍所在地和现居住地计划生育信息共享，并与相关部门有关人口的信息管理系统实现信息共享。

　　县级以上地方人民政府人口和计划生育部门主管本行政区域内流动人口计划生育工作，落实本级人民政府流动人口计划生育管理和服务措施；组织实施流动人口计划生育工作检查和考核；建立流动人口计划生育信息通报制度，汇总、通报流动人口计划生育信息；受理并及时处理与流动人口计划生育工作有关的举报，保护流动人口相关权益。

　　县级以上人民政府公安、民政、人力资源社会保障、住房城乡建设、卫生、价格等部门和县级以上工商行政管理部门在各自职责范围内，负责有关的流动人口计划生育工作。

　　**第六条**　乡（镇）人民政府、街道办事处负责本管辖区域内流动人口计划生育工作，对流动人口实施计划生育管理，开展计划生育宣传教育；组织从事计划生育技术服务的机构指导流动人口中的育龄夫妻（以下称育龄夫妻）选择安全、有效、适宜的避孕节育措施，依法向育龄夫妻免费提供国家规定的基本项目的计划生育技术服务。

　　流动人口现居住地和户籍所在地的乡（镇）人民政府、街道办事处之间建立流动人口计划生育信息通报制度，及时采集流动人口计划生育信息，运用流动人口计划生育信息管理系统核实、通报流动人口计划生育信息。

　　**第七条**　流动人口中的成年育龄妇女（以下称成年育龄妇女）在离开户籍所在地前，应当凭本人居民身份证到户籍所在地的乡（镇）人民政府或者街道办事处办理婚育证明；已婚的，办理婚育证明还应当出示结婚证。婚育证明应当载明成年育龄妇女的姓名、年龄、公民身份号码、婚姻状况、配偶信息、生育状况、避孕节育情况等内容。

　　流动人口户籍所在地的乡（镇）人民政府、街道办事处应当及时出具婚育证明。

　　**第八条**　成年育龄妇女应当自到达现居住地之日起30日内提交婚育证明。成年育龄妇女可以向现居住地的乡（镇）人民政府或者街道办事处提交婚育证明，也可以通过村民委员会、居民委员会向现居住地的乡（镇）人民政府或者街道办事处提交婚育证明。

　　流动人口现居住地的乡（镇）人民政府、街道办事处应当查验婚育证明，督促未办理婚育证明的成年育龄妇女及时补办婚育证明；告知流动人口在现居住地可以享受的计划生育服务和奖励、优待，以及应当履行的计划生育相关义务。

　　村民委员会、居民委员会应当协助乡（镇）人民政府、街道办事处开展本条第二款规定的工作，做好流动人口婚育情况登记。

　　**第九条**　流动人口现居住地的县级人民政府公安、民政、人力资源社会保障、卫生等部门和县级工商行政管理部门应当结合部门职责，将流动人口计划生育工作纳入相关管理制度；及时向所在地同级人口和计划生育部门通报在办理有关登记和证照等工作中了解的流动人口婚育证明办理情况等计划生育信息。

　　接到通报的人口和计划生育部门应当及时会同乡（镇）人民政府、街道办事处落实流动人口计划生育管理和服务措施。

　　**第十条**　流动人口在现居住地享受下列计划生育服务和奖励、优待：

　　（一）免费参加有关人口与计划生育法律知识和生殖健康知识普及活动；

　　（二）依法免费获得避孕药具，免费享受国家规定的其他基本项目的计划生育技术服务；

　　（三）晚婚晚育或者在现居住地施行计划生育手术的，按照现居住地省、自治区、直辖市或者较大的市的规定，享受休假等；

　　（四）实行计划生育的，按照流动人口现居住地省、自治区、直辖市或者较大的市的规定，在生产经营等方面获得支持、优惠，在社会救济等方面享受优先照顾。

　　**第十一条**　流动人口现居住地的地方各级人民政府和县级以上地方人民政府有关部门应当采取措施，落实本条例第十条规定的流动人口计划生育服务和奖励、优待。

　　流动人口户籍所在地的地方各级人民政府和县级以上地方人民政府有关部门应当依法落实法律、法规和规章规定的流动人口计划生育服务和奖励、优待。

　　**第十二条**　育龄夫妻应当自觉落实计划生育避孕节育措施，接受户籍所在地和现居住地人民政府的计划生育管理。

　　**第十三条**　流动人口现居住地从事计划生育技术服务的机构应当按照所在地省、自治区、直辖市或者较大的市的规定，为已婚育龄妇女出具避孕节育情况证明。

　　流动人口现居住地的乡（镇）人民政府或者街道办事处应当根据已婚育龄妇女的避孕节育情况证明，及时向其户籍所在地的乡（镇）人民政府或者街道办事处通报流动人口避孕节育情况。流动人口户籍所在地的县级人民政府人口和计划生育部门、乡（镇）人民政府或者街道办事处不得要求已婚育龄妇女返回户籍所在地进行避孕节育情况检查。

　　**第十四条**　流动人口现居住地的村民委员会、居民委员会应当协助所在地的乡（镇）人民政府或者街道办事处了解本村或者本居住地区流动人口计划生育情况，及时向乡（镇）人民政府或者街道办事处通报相关信息。

　　房屋租赁中介机构、房屋的出租（借）人和物业服务企业等有关组织和个人在村民委员会、居民委员会了解流动人口计划生育情况时，应当如实提供相关信息。

　　**第十五条**　用人单位应当做好本单位流动人口计划生育工作，依法落实法律、法规和规章规定的流动人口计划生育奖励、优待，接受所在地的乡（镇）人民政府或者街道办事处和县级以上地方人民政府人口和计划生育部门的监督、检查。

　　**第十六条**　育龄夫妻生育第一个子女的，可以在现居住地的乡（镇）人民政府或者街道办事处办理生育服务登记。办理生育服务登记，应当提供下列证明材料：

　　（一）夫妻双方的居民身份证；

　　（二）结婚证；

　　（三）女方的婚育证明和男方户籍所在地的乡（镇）人民政府或者街道办事处出具的婚育情况证明材料。

　　育龄夫妻现居住地的乡（镇）人民政府或者街道办事处应当自收到女方的婚育证明和男方的婚育情况证明材料之日起7个工作日内，向育龄夫妻户籍所在地的乡（镇）人民政府或者街道办事处核实有关情况。育龄夫妻户籍所在地的乡（镇）人民政府或者街道办事处应当自接到核实要求之日起15个工作日内予以反馈。核查无误的，育龄夫妻现居住地的乡（镇）人民政府或者街道办事处应当在接到情况反馈后即时办理生育服务登记；情况有误、不予办理的，应当书面说明理由。

　　现居住地的乡（镇）人民政府或者街道办事处应当自办理生育服务登记之日起15个工作日内向育龄夫妻户籍所在地的乡（镇）人民政府或者街道办事处通报办理结果。

　　**第十七条**　出具婚育证明或者其他计划生育证明材料，不得收取任何费用。

　　流动人口计划生育工作所需经费，按照国家有关规定予以保障。

　　**第十八条**　地方各级人民政府和政府有关部门以及协助查验婚育证明的村民委员会、居民委员会及其工作人员，应当对涉及公民隐私的流动人口信息予以保密。

　　**第十九条**　县级以上人民政府人口和计划生育部门未依照本条例的规定履行流动人口计划生育工作职责的，由本级人民政府或者上级人民政府人口和计划生育部门责令改正，通报批评；情节严重的，对主要负责人、直接负责的主管人员和其他直接责任人员依法给予处分。

　　**第二十条**　流动人口户籍所在地的乡（镇）人民政府或者街道办事处在流动人口计划生育工作中有下列情形之一的，分别由乡（镇）人民政府的上级人民政府或者设立街道办事处的人民政府责令改正，通报批评；情节严重的，对主要负责人、直接负责的主管人员和其他直接责任人员依法给予处分：

　　（一）未依照本条例规定为流动人口出具计划生育证明材料，出具虚假计划生育证明材料，或者出具计划生育证明材料收取费用的；

　　（二）违反本条例规定，要求已婚育龄妇女返回户籍所在地进行避孕节育情况检查的；

　　（三）未依法落实流动人口计划生育奖励、优待的；

　　（四）未依照本条例规定向流动人口现居住地的乡（镇）人民政府、街道办事处反馈流动人口计划生育信息的；

　　（五）违反本条例规定的其他情形。

　　**第二十一条**　流动人口现居住地的乡（镇）人民政府或者街道办事处在流动人口计划生育工作中有下列情形之一的，分别由乡（镇）人民政府的上级人民政府或者设立街道办事处的人民政府责令改正，通报批评；情节严重的，对主要负责人、直接负责的主管人员和其他直接责任人员依法给予处分：

　　（一）未依照本条例规定向育龄夫妻免费提供国家规定的基本项目的计划生育技术服务，或者未依法落实流动人口计划生育奖励、优待的；

　　（二）未依照本条例规定查验婚育证明的；

　　（三）未依照本条例规定为育龄夫妻办理生育服务登记，或者出具虚假计划生育证明材料，或者出具计划生育证明材料收取费用的；

　　（四）未依照本条例规定向流动人口户籍所在地的乡（镇）人民政府、街道办事处通报流动人口计划生育信息的；

　　（五）违反本条例规定的其他情形。

　　**第二十二条**　流动人口现居住地的县级人民政府公安、民政、人力资源社会保障、卫生等部门和县级工商行政管理部门违反本条例第九条规定的，由本级人民政府或者上级人民政府主管部门责令改正，通报批评。

　　**第二十三条**　流动人口未依照本条例规定办理婚育证明的，现居住地的乡（镇）人民政府或者街道办事处应当通知其在3个月内补办；逾期仍不补办或者拒不提交婚育证明的，由流动人口现居住地的乡（镇）人民政府或者街道办事处予以批评教育。

　　**第二十四条**　用人单位违反本条例第十五条规定的，由所在地县级人民政府人口和计划生育部门责令改正，通报批评。

　　房屋租赁中介机构、房屋的出租（借）人和物业服务企业等有关组织或者个人未依照本条例规定如实提供流动人口信息的，由所在地的乡（镇）人民政府或者街道办事处责令改正，予以批评教育。

　　**第二十五条**　本条例自2009年10月1日起施行。1998年8月6日国务院批准、1998年9月22日原国家计划生育委员会发布的《[流动人口计划生育工作管理办法](https://www.pkulaw.com/chl/c8062869d0fb3652bdfb.html)》同时废止。

© 北大法宝：（[www.pkulaw.com](https://www.pkulaw.com/)) 专业提供法律信息、法学知识和法律软件领域各类解决方案。北大法宝为您提供丰富的参考资料，正式引用法规条文时请与标准文本核对。
欢迎查看所有[产品和服务](https://www.pkulaw.com/helps/2.html)。法宝快讯：[如何快速找到您需要的检索结果？](https://www.pkulaw.com/helps/70.html)[法宝](https://www.pkulaw.com/helps/69.html)V6有何新特色？

扫描二维码阅读原文

**原文链接：**<https://www.pkulaw.com/chl/f6b2c35d55b7a40ebdfb.html>
